# Supplementary material for: The Loss of PPARγ Expression and Signaling Is a Key Feature of Cutaneous Actinic Disease and Squamous Cell Carcinoma: Association with Tumor Stromal Inflammation
Source: Cells. 2024 Aug 15;13(16):1356. doi: 10.3390/cells13161356 (PMC11352891; doi:10.3390/cells13161356)
Supplement: Supplementary file 1 [file cells-13-01356-s001.zip › Figure S2.pdf]

Figure S2: Single Cell RNA Sequencing Data Summary for *Pparg*-/-^{epi} (KO) mouse skin.

10,228
Estimated Number of Cells

49,564
Mean Reads per Cell

2,989
Median Genes per Cell

Sequencing

Number of Reads	506,940,549
Number of Short Reads Skipped	0
Valid Barcodes	97.7%
Valid UMIs	100.0%
Sequencing Saturation	52.5%
Q30 Bases in Barcode	95.8%
Q30 Bases in RNA Read	93.7%
Q30 Bases in UMI	95.3%

Mapping

Reads Mapped to Genome	95.9%
Reads Mapped Confidently to Genome	92.5%
Reads Mapped Confidently to Intergenic Regions	4.9%
Reads Mapped Confidently to Intronic Regions	19.7%
Reads Mapped Confidently to Exonic Regions	67.9%
Reads Mapped Confidently to Transcriptome	65.1%
Reads Mapped Antisense to Gene	1.6%

Cells

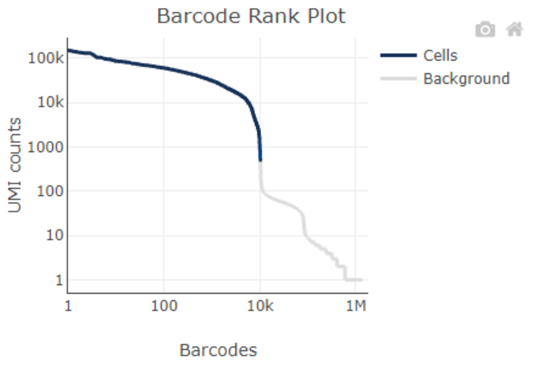

Estimated Number of Cells	10,228
Fraction Reads in Cells	96.2%
Mean Reads per Cell	49,564
Median UMI Counts per Cell	11,765
Median Genes per Cell	2,989
Total Genes Detected	23,797

Sample

Sample ID	KO
Sample Description	
Chemistry	Single Cell 3' v3
Include introns	False
Reference Path	…index/v4.0.0/refdata-gex-mm10-2020-A
Transcriptome	mm10-2020-A
Pipeline Version	cellranger-6.1.1
