# Supplementary material for: The Loss of PPARγ Expression and Signaling Is a Key Feature of Cutaneous Actinic Disease and Squamous Cell Carcinoma: Association with Tumor Stromal Inflammation
Source: Cells. 2024 Aug 15;13(16):1356. doi: 10.3390/cells13161356 (PMC11352891; doi:10.3390/cells13161356)
Supplement: Supplementary file 1 [file cells-13-01356-s001.zip › Figure S3.pdf]

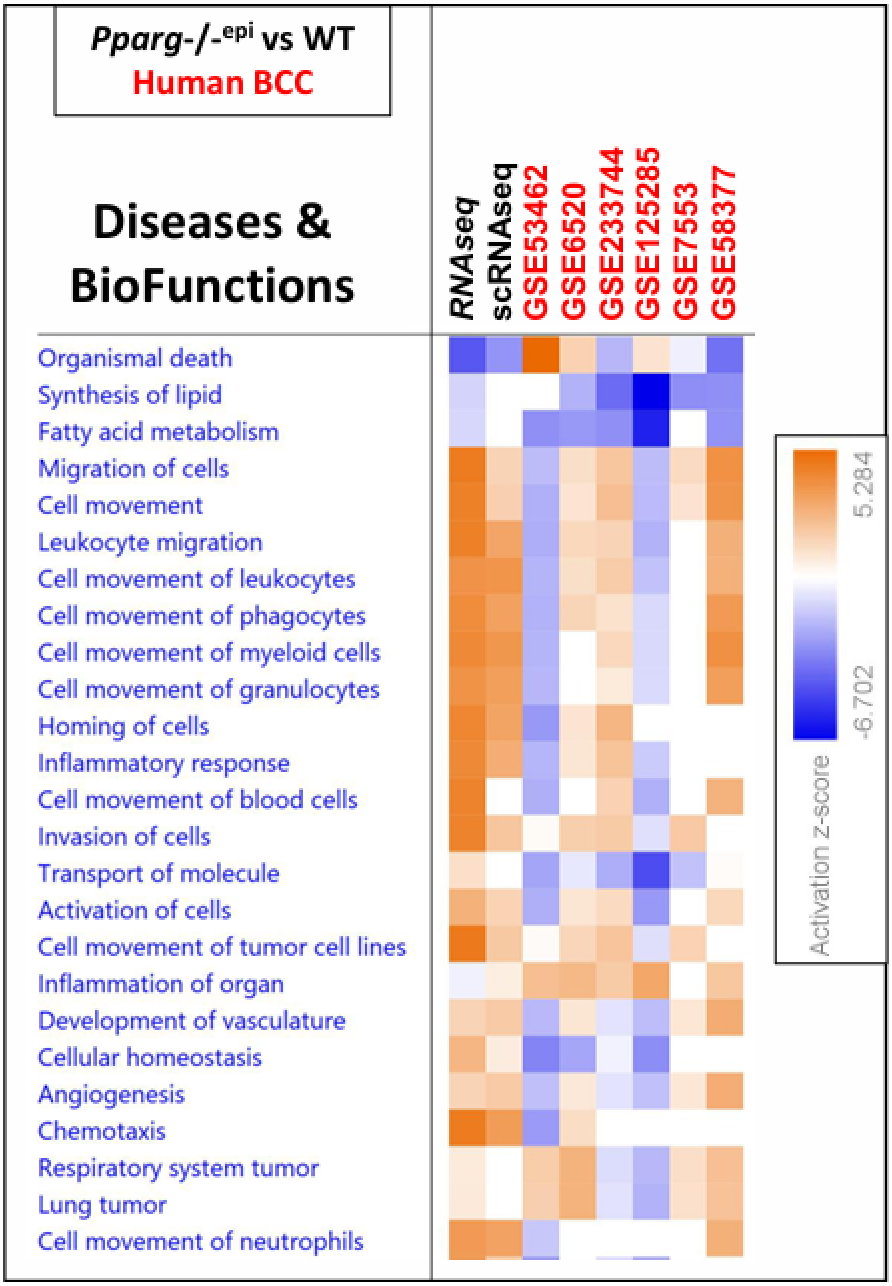

Figure S3: Top Diseases and Biofunctions that are common to *Pparg*-/-^{epi} mice and human BCC. After uploading DEG data for *Pparg*-/-^{epi} mouse whole transcriptomic RNA sequencing (RNAseq), *Pparg*-/-^{epi} single cell RNA sequencing (scRNAseq), and BCC datasets to Qiagen's Ingenuity Pathway Analysis, a comparison analysis was performed for Diseases and Biofunctions. The heat map shows the top 25 Diseases and Biofunctions that are common to *Pparg*-/-^{epi} mouse skin and BCCs (ranked by z-score).
