# Supplementary material for: The Loss of PPARγ Expression and Signaling Is a Key Feature of Cutaneous Actinic Disease and Squamous Cell Carcinoma: Association with Tumor Stromal Inflammation
Source: Cells. 2024 Aug 15;13(16):1356. doi: 10.3390/cells13161356 (PMC11352891; doi:10.3390/cells13161356)
Supplement: Supplementary file 1 [file cells-13-01356-s001.zip › Figure S5.pdf]

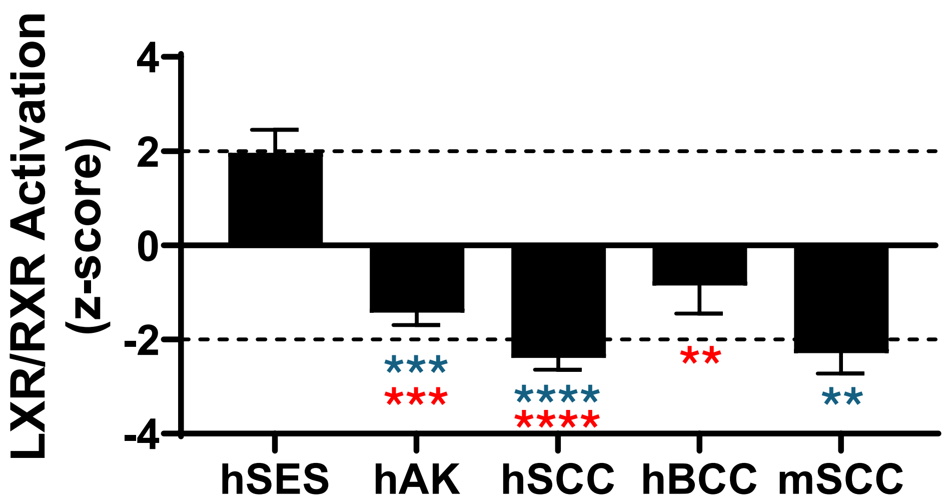

Figure S5: Canonical Pathway analysis shows tumor-specific reduction in LXR/RXR activation. Transcriptomic datasets were obtained for human sun-exposed skin (hSES) relative to non-sun exposed skin. In addition, transcriptomic datasets that compared human and mouse tumors with non-tumor control skin was obtained (Table S3). These datasets included human actinic keratoses (hAK), human cutaneous squamous cell carcinomas (hSCC), human basal cell carcinomas (hBCC), and mouse cutaneous squamous cell carcinoma (mSCC) were also obtained. After uploading the DEGs from each dataset to Qiagen's Ingenuity Pathway Analysis, activation z-scores for the canonical pathway LXR/RXR activation were obtained. The plot shows the mean and SEM of the activation z-scores. Red asterisk* = For human tumors: Significantly different from hSES expression (1-way ANOVA). Blue asterisks* = Significantly different from zero (One sample T-test). **, p<0.01; ***, p<0.001; ****, p<0.0001.
