# Supplementary material for: The Loss of PPARγ Expression and Signaling Is a Key Feature of Cutaneous Actinic Disease and Squamous Cell Carcinoma: Association with Tumor Stromal Inflammation
Source: Cells. 2024 Aug 15;13(16):1356. doi: 10.3390/cells13161356 (PMC11352891; doi:10.3390/cells13161356)
Supplement: Supplementary file 1 [file cells-13-01356-s001.zip › Table S3.pdf]

Table S3: Cluster identification

Enrichr Cluster Cell ID (Non-keratinocyte, non-CD45+)					Cell #/genotype and % of non-Keratinocyte			
**Cluster**	**Identified Cell Type**	**Overlap**	**Adjusted P-value**	**Combined Score**	***Pparg-/-*** **epi**	**%**	**WT**	**%**
1	Fibroblasts	26/232	3.94E-21	1036.57	909	13.05%	2878	40.38%
3	Fibroblasts	33/232	8.26E-31	2063.53	311	4.47%	414	5.81%
	Pericytes	25/155	1.24E-24	1795.36				
4	Melanocytes	13/133	3.43E-09	382.06	301	4.32%	231	3.24%
	Smooth Muscle Cells	12/169	4.17E-07	207.19				
	Pericytes	11/155	9.54E-07	189.55				
	Mast Cells	13/235	9.54E-07	146.6				
5	Fibroblasts	33/232	9.00E-31	2063.53	182	2.61%	349	4.90%
9	Fibroblasts	24/232	1.40E-18	826.03	187	2.69%	288	4.04%
	Endothelial Cells (Aorta)	17/185	2.88E-12	459.61				
10	Adipocytes	33/210	3.05E-32	2430.03	219	3.14%	212	2.97%
	Fibroblasts	33/232	4.70E-31	2063.53				
16	Pancreatic Stellate Cells	16/111	2.10E-14	897.99	193	2.77%	16	0.22%
	Fibroblasts	18/232	8.21E-12	369.17				
18	Smooth Muscle Cells	33/169	1.25E-35	3480.92	25	0.36%	55	0.77%
20	Fibroblasts	48/232	1.70E-54	6471.63	1025	14.72%	379	5.32%
	Pancreatic Stellate Cells	39/111	3.73E-54	12275.53				
21	Fibroblasts	28/232	8.66E-24	1281.13	540	7.75%	850	11.93%
				**Sum**	**3892**	**55.89%**	**5672**	**79.58%**

Enrichr Cluster Cell ID (CD45(+)/CD3(-))					Cell #/genotype and % of non-Keratinocyte			
**Cluster**	**Identified Cell Type**	**Overlap**	**Adjusted P-value**	**Combined Score**	***Pparg-/-*** **epi**	**% Total**	**WT**	**% Total**
6	Neutrophils	51/151	8.68E-71	16862.98	488	7.01%	6	0.08%
7	Macrophages	54/204	1.47E-68	11869.25	293	4.21%	191	2.68%
	Microglia	45/158	2.66E-58	10206.96				
	Monocytes	46/176	6.89E-58	9057.66				
11	Langerhans Cells	22/123	2.68E-22	1830.78	250	3.59%	165	2.32%
14	Dendritic Cells	47/199	1.70E-56	7848.04	157	2.25%	177	2.48%
	Macrophages	46/204	1.76E-54	7022.2				
22	Macrophages	60/204	4.44E-80	17071.57	694	9.97%	457	6.41%
	Myeloid-derived Suppressor Cells	43/112	2.99E-62	16748.92				
			**CD45(+)/CD3(-) Sum**		**1882**	**27.02%**	**996**	**13.98%**

Table S3 (Continued)

Enrichr Cluster Cell ID (CD45(+)/CD3(+))					Cell #/genotype and % of non-Keratinocyte			
**Cluster**	**Identified Cell Type**	**Overlap**	**Adjusted P-value**	**Combined Score**	***Pparg*-/-epi**	**%**	**WT**	**%**
12	T Cells	43/165	2.63E-53	8166.93	220	3.16%	185	2.60%
	T Memory Cells	40/133	1.61E-52	9515.05				
15	T Cells	61/165	8.56E-89	26960.51	227	3.26%	101	1.42%
	NK Cells	59/157	1.83E-86	26281.46				
	T Memory Cells	54/133	4.51E-81	26507.54				
	Natural Killer T Cells	47/118	1.29E-69	20546.75				
24	T Cells	60/165	1.07E-86	25364.92	743	10.67%	173	2.43%
	T Memory Cells	53/133	9.10E-79	24760.25				
			**CD45(+)/CD3(+) Sum**		**1190**	**17.09%**	**459**	**6.44%**
			**CD45+ Sum**		**3072**	**44.11%**	**1455**	**20.42%**

Enrichr Cluster Cell ID (Keratinocytes)					Cell #/genotype and % of Keratinocyte			
**Cluster**	**Identified Cell Type**	**Overlap**	**Adjusted P-value**	**Combined Score**	***Pparg*-/-epi**	**%**	**WT**	**%**
2	Keratinocytes	19/127	1.58E-17	1154.35	652	31.67%	962	22.06%
8	Epithelial Cells	25/218	1.78E-17	626.1	295	14.33%	187	4.29%
	Basal Cells	11/141	5.82E-06	136.77				
	Keratinocytes	10/127	1.47E-05	126.77				
13	Keratinocytes	23/127	1.68E-23	1968.58	150	7.29%	1301	29.84%
	Basal Cells	23/141	1.07E-22	1656.79				
17	Keratinocytes	34/127	6.27E-42	6205.59	36	1.75%	84	1.93%
19	Sebocytes	5/124	0.059469	34.39	0	0.00%	18	0.41%
23	Keratinocytes	36/127	1.28E-45	7407.51	310	15.06%	806	18.49%
25	Keratinocytes	15/127	5.11E-12	605.47	222	10.78%	666	15.28%
26	Keratinocytes	29/127	3.37E-33	3844.71	394	19.14%	336	7.71%
				**Sum**	**2059**	**100.0%**	**4360**	**100.0%**
