# Supplementary material for: The Loss of PPARγ Expression and Signaling Is a Key Feature of Cutaneous Actinic Disease and Squamous Cell Carcinoma: Association with Tumor Stromal Inflammation
Source: Cells. 2024 Aug 15;13(16):1356. doi: 10.3390/cells13161356 (PMC11352891; doi:10.3390/cells13161356)
Supplement: Supplementary file 1 [file cells-13-01356-s001.zip › Table S4.pdf]

Table S4: Published Transcriptomic datasets used for analysis

Mouse cSCC vs Normal				
**STUDY**	**SAMPLE TYPE**	***Ppar* Expression**<br>**Log2FC (Adj p-value) or (Exp p-value)***	**METHODOLOGY**	**REF**
GSE84292	SCC (n=6)<br>SES (n=6)	*Ppara* = -1.240 (2.61E-04)<br>*Ppard* = 1.034 (2.90E-04)<br>*Pparg* = -2.358 (2.85E-05)	UV-induced mouse tumors. Whole transcriptome, Illumina HiSeq 2000	[72]
GSE63967	SCC (n=10)<br>Control (n=9)	*Ppara* = -1.944 (4.39E-07)<br>*Ppard* = 1.186 (6.77E-06)<br>*Pparg* = -2.297 (1.41E-06)	DMBA/TPA-induced tumors and matched dorsal skin. Affymetrix Mouse Gene 1.1 ST Array	[73]
GSE63967	Spindle SCC (n=6)<br>Control (n=9)	*Ppara* = -2.100 (4.92E-08)*<br>*Ppard* = 0.380 (9.21E-02)*<br>*Pparg* = --1.810 (1.35E-05)*	DMBA/TPA-induced tumors and matched dorsal skin. Affymetrix Mouse Gene 1.1 ST Array	[73]
GSE19616	SCC (n=5)<br>Control (n=4)	*Ppara* = -2.346 (1.67E-05)*<br>*Ppard* = 0.085 (5.25E-01)*<br>*Pparg* = -2.675 (2.42E-06)*	Spontaneous tumors: *Rb* & *Tp53* epidermal specific knockout (Testing dataset). Affymetrix Mouse Genome 430 2.0 Array	[74]
GSE19616	SCC (n=7)<br>Control (n=4)	*Ppara* = -2.690 (3.69E-05)*<br>*Ppard* = 0.377 (3.66E-01)*<br>*Pparg* = -2.830 (5.43E-08)*	Spontaneous tumors: *Tp53* epidermal specific knockout (Testing dataset).<br>Affymetrix Mouse Genome 430 2.0 Array	[74]
GSE11990	SCC (n=8)<br>Control (n=5)	*Ppara* = -1.860 (1.23E-02)<br>*Ppard* = 1.351 (3.93E-02)<br>*Pparg* = -1.932 (5.20E-02)	Spontaneous tumors: *Rb* & *Tp53* epidermal specific knockout (Training dataset).<br>Affymetrix Mouse Genome 430 2.0 Array	[74]
GSE11990	SCC (n=7)<br>Control (n=5)	*Ppara* = -2.221 (1.27E-04)	Spontaneous tumors: *Tp53* epidermal specific knockout (Training dataset).	[74]

		Ppard = 0.566 (2.14E-01)<br>*Pparg* = -2.859 (3.49E-06)	Affymetrix Mouse Genome 430 2.0 Array	
GSE89462	cSCC (n=3)<br>Control (n=3)	*Ppara* = -1.240 (2.61E-04)<br>*Ppard* = 1.034 (2.90E-04)<br>*Pparg* = -4.877 (8.02E-04)	Mouse DMBA/PMA-induced SCC<br>Agilent-028005 SurePrint G3 Mouse GE 8x60K Microarray	[75]
**Sun-exposed skin (SES) vs Non-exposed skin (NES)**				
**STUDY**	**SAMPLE TYPE**	***PPAR* Expression**<br>**Log2FC (Adj p-value) or (Exp p-value)*-**	**METHODOLOGY**	**REF**
GSE98774	SES (n=20)<br>NES (n=16)	*PPARA* = 0.267 (5.08E-01)<br>*PPARD* = -0.248 (5.67E-01)<br>*PPARG* = 0.583 (3.50E-01)	Affymetrix Human Genome U133 Plus 2.0 Array	UN
GSE142108	SES (n=15)<br>NES (n=15)	*PPARA* = 0.145 (1.00E00)<br>*PPARD* = -0.028 (1.00E00)<br>*PPARG* = 0.874 (9.52E-01)	Affymetrix Clariom S Assay, Human	[76]
Kita & Fraser, 2016	SES (n=302)<br>NES (n=196)	*PPARA = ND*<br>*PPARD = ND*<br>*PPARG = ND*	Analysis of GTEX portal data. DEGs analyzed by DESeq2. Report 522 genes with > 2-fold change and FDR < 0.01.	[34]
Genotype Tissue Expression	SES: lower leg (n=700)<br>NSES: Supra-pubic (n=604)	*PPARA* = 0.232 (2.20E-04)<br>*PPARD* = 0.195 (5.57E-04)<br>*PPARG* = 0.303 (3.02E-06)	Tissue-specific TPM downloaded [www.gtexportal.org]. DEG calculated using edgeR (DEApp website: yanli.shinyapps.io/DEApp/)	[28]
Zou *et al* 2021	SES: (n=6)<br>NSES: (n=6)	*PPARA = ND*<br>*PPARD = ND*<br>*PPARG = ND*	Illumina HiSeq 4000 with 150 bp paired-end reads. DEGs defined as genes with |log2Fold change|>2, FDR <0.05, and Padj <0.05.	[35]
**Human Actinic Keratosis (AK) vs Normal skin**				
**STUDY**	**SAMPLE TYPE**	***PPAR* Expression**<br>**Log2FC (Adj p-value) or (Exp p-value)***	**METHODOLOGY**	**REF**

E-MTAB-5678	AK (n=13)<br>NES (n=4)	*PPARA* = -0.200 (7.12E-01)*<br>*PPARD* = 3.800 (8.99E-36)*<br>*PPARG* = -1.400 (1.74E-03)*	Whole transcriptome,<br>RNAseq (500 ng), Illumina HiSeq 2500, (100 bp, paired-end)	[77]
GSE98774	AK (n=18)<br>SES (n=20)	*PPARA* = -0.217 (3.87E-01)<br>*PPARD* = 1.045 (6.08E-05)<br>*PPARG* = -0.304 (5.87E-01)	Affymetrix Human Genome U133 Plus 2.0 Array	UN
GSE84293	AK (n=10)<br>SES (n=7)	*PPARA* = -0.732 (5.00E-01)<br>*PPARD* = 0.018 (1.00E00)<br>*PPARG* = -1.003 (6.66E-01)	Whole transcriptome, Illumina HiSeq 2000	[72]
Bailey et al, 2023	AK (n=14)<br>SES (n=26)	*PPARA* = -0.252 (3.15E-01)<br>*PPARD* = 1.102 (3.96E-05)<br>*PPARG* = -0.399 (5.69E-01)	Whole transcriptome, RNAseq (0.5 – 1.0 µg), Illumina HISeq2000	[78]
GSE2503	AK (n=5)<br>Normal (n=5)	*PPARA* = -0.955 (5.23E-01)<br>*PPARD* = 1.889 (2.64E-01)<br>*PPARG* = -3.145 (1.72E-01)	Affymetrix Human Genome U133A Array	[79]
GSE32628	AK (n=14)<br>SES (n=13)	*PPARA* = 0.005 (6.81E-01)<br>*PPARD* = 0.161 (2.80E-04)<br>*PPARG* = 0.364 (3.44E-03)	Illumina Human WG6 v2 Expression BeadChips	[80]
GSE108008	AK (n=10)<br>SES (n=10)	*PPARA* = 0.046 (1.00E00)<br>*PPARD* = 0.357 (1.00E00)<br>*PPARG* = 0.247 (1.00E00)	Affymetrix GeneChip Human Gene 2.0 ST Array	[81]

GSE142108	AK (n=15)<br>NES (n=15)	*PPARA* = 0.052 (8.85E-01)<br>*PPARD* = 0.285 (6.42E-01)<br>*PPARG* = 0.489 (6.99E-01)	Affymetrix Clariom S Assay, Human	[76]
**Human cutaneous squamous cell carcinoma (cSCC) vs Normal skin**				
**STUDY**	**SAMPLE TYPE**	***PPAR* Expression**<br>**Log2FC (Adj p-value) or (Exp p-value)***	**METHODOLOGY**	**REF**
E-MTAB-5678	cSCC (n=5)<br>NES (n=4)	*PPARA* = -0.100 (8.23E-01)*<br>*PPARD* = 3.300 (3.45E-41)*<br>*PPARG* = -0.200 (5.63E-01)*	Whole transcriptome,<br>RNAseq (500 ng), Illumina HiSeq 2500, (100 bp, paired-end)	[77]
GSE84293	cSCC (n=9)<br>SES (n=7)	*PPARA* =-1.233 (7.04E-03)<br>*PPARD* = 0.104 (9.49E-01)<br>*PPARG* = -2.289 (9.84E-05)	Illumina HiSeq 2000 (Homo sapiens)	[72]
Hu et al 2022	cSCC (n=3)<br>Adjacent SES (n=3)	*PPARA* = -1.684 (2.68E-01)*<br>*PPARD** = 1.128 (6.94E-01)*<br>*PPARG* = -1.598 (4.66E-01)*	mRNA & lncRNA array. CapitalBio Technology Human lncRNA Array V4	[82]
Bailey et al, 2023	cSCC (n=66)<br>SES (n=26)	*PPARA* = -0.960 (6.75E-13)<br>*PPARD* = 1.043 (1.043E-11)<br>*PPARG* = -2.234 (1.23E-10)	Whole transcriptome, RNAseq (0.5 – 1.0 µg), Illumina HISeq2000	[78]
GSE139505	cSCC (n=9)<br>Normal skin (n=7)	*PPARA* = -0.369 (1.84E-01)<br>*PPARD* = 1.244 (2.13E-09)<br>*PPARG* = -1.134 (8.77E-02)	Whole transcriptome, RNAseq, Illumina NextSeq 500 (Homo sapiens)	[83]
GSE108008	cSCC (n=10)<br>SES (n=10)	*PPARA* = -0.117 (6.78E-01)	Affymetrix GeneChip Human Gene 2.0 ST Array	[81]

		PPARD = 0.734 (1.06E-02)<br>*PPARG* = -0.818 (1.39E-03)		
GSE125285	SCC (n=10)<br>Adjacent SES (n=10)	*PPARA* = -0.284 (5.52E-01)<br>*PPARD* = 0.767 (1.70E-03)<br>*PPARG* = -2.791 (3.89E-05)	Illumina HiSeq 2000 (Homo sapiens)	[84]
GSE2503	cSCC (n=5)<br>SES (n=5)	*PPARA* = 0.168 (8.88E-01)<br>*PPARD* = 1.447 (7.15E-02)<br>*PPARG* = -2.579 (1.38E-01)	cDNA, Affymetrix Human Genome U133A Array	[79]
GSE45164	cSCC (n=10)<br>Normal (n=3)	*PPARA* = -0.125 (6.08E-01)<br>*PPARD* = 0.551 (2.78E-01)<br>*PPARG* =-0.299 (4.65E-01)	cDNA, Affymetrix Human Genome U133A 2.0 Array	[85]
GSE32628	cSCC (n=15)<br>NES (n=13)	*PPARA* = -0.011 (7.49E-01)<br>*PPARD* = 0.435 (1.39E-06)<br>*PPARG* = 0.265 (1.65E-02)	cDNA (100 ng), Illumina Human WG6 v2 Expression BeadChips	[80]
Haider *et al*, 2006	cSCC (n=8)<br>SES (n=8)	*PPARA* = ND<br>*PPARD* = 0.664 (5.00E-02)*<br>*PPARG* = -1.155 (5.00E-02)*	Affymetrix U95A-set GeneChip probe<br>array	[86]
GSE7553	cSCC (n=11)<br>Normal (n=4)	*PPARA* = -1.040 (9.70E-02)<br>*PPARD* = 0.954 (7.26E-02)<br>*PPARG* = -3.000 (1.36E-01)	Affymetrix Human Genome U133 Plus 2.0 Array	[87]
GSE53462	SCC (n=4)<br>Normal (n=5)	*PPARA* = -0.323 (1.53E-01)<br>*PPARD* = -0.350 (1.43E-01)	Illumina HiSeq 2000 (Homo sapiens)	[88]

		PPARG = -0.466 (4.99E-01)		
**Basal Cell Carcinoma**				
**STUDY**	**SAMPLE TYPE**	***PPAR* Expression Log2FC (Adj p-value) or (Exp p-value)*-**	**METHODOLOGY**	**REF**
GSE125285	BCC (n=25)<br>Adjacent SES (n=25)	*PPARA* = -0.557 (9.60E-04)<br>*PPARD* = -0.504 (2.14E-07)<br>*PPARG* = -2.77 (2.38E-16)	Illumina HiSeq 2000 (Homo sapiens)	[84]
GSE7553	BCC (n=15)<br>Normal (n=4)	*PPARA* = -0.631 (2.08E-01)<br>*PPARD* = 1.060 (2.29E-01)<br>*PPARG* = -3.088 (6.62E-03)	Affymetrix Human Genome U133 Plus 2.0 Array	[87]
GSE6520	BCC (n=23)<br>Normal (n=8)	*PPARA* = ND<br>*PPARD* = ND<br>*PPARG* = ND	3 subtypes of BCC [superficial (n=8), nodular (n=8), morphoeic (n=7)] included.	[89]
GSE53462	BCC (n=16)<br>Normal (n=5)	*PPARA* = -0.437 (1.33E-01)<br>*PPARD* = -0.535 (1.77E-02)<br>*PPARG* = -0.510 (6.16E-01)	Illumina HumanHT-12 V4.0 expression beadchip	[88]
GSE233744	BCC (n=9)<br>Normal (n=9)	*PPARA* = ND<br>*PPARD* = ND<br>*PPARG* = -4.231 (3.13E-09)	Illumina NovaSeq 6000 (Homa sapiens). Reporting cutoffs (Fold change >2, p-value <0.05).	UN
GSE58377	BCC (n=13)<br>Normal (n=8)	*PPARA* = ND<br>*PPARD* = ND<br>*PPARG* = -2.607 (1.64E-15)	Illumina HiSeq 2500: paired-end at 100 bp. Reporting cutoffs (Log2FC > Abs (1.0), FDR <0.05).	[90]
**Epidermis Only**				
**STUDY**	**SAMPLE TYPE**	***PPAR* Expression Log2FC (Adj p-value) or (Exp p-value)*-**	**METHODOLOGY**	**REF**

GSE42677	Human SCC (n=10)<br>Normal Epidermis (n=10)	*PPARA* = -1.582 (3.61E-08)<br>*PPARD* = 1.501 (1.62E-04)<br>*PPARG* = -1.262 (6.84E-11)	Laser microdissection: cDNA, Affymetrix Human Genome U133A 2.0 Array	[36]
GSE42677	Human AK (n=5)<br>Normal Epidermis (n=10)	*PPARA* = -1.543 (5.65E-05))<br>*PPARD* = 1.308 (6.43E-05)<br>*PPARG* = -1.014 (6.59E-04)	Laser microdissection: cDNA, Affymetrix Human Genome U133A 2.0 Array	[36]
UN = unpublished				
