# Supplementary figures and images for: The Loss of PPARγ Expression and Signaling Is a Key Feature of Cutaneous Actinic Disease and Squamous Cell Carcinoma: Association with Tumor Stromal Inflammation
Source: Cells. 2024 Aug 15;13(16):1356. doi: 10.3390/cells13161356 (PMC11352891; doi:10.3390/cells13161356)

Figure S1: Single Cell RNA Sequencing Data Summary for Wildtype (WT) mouse skin

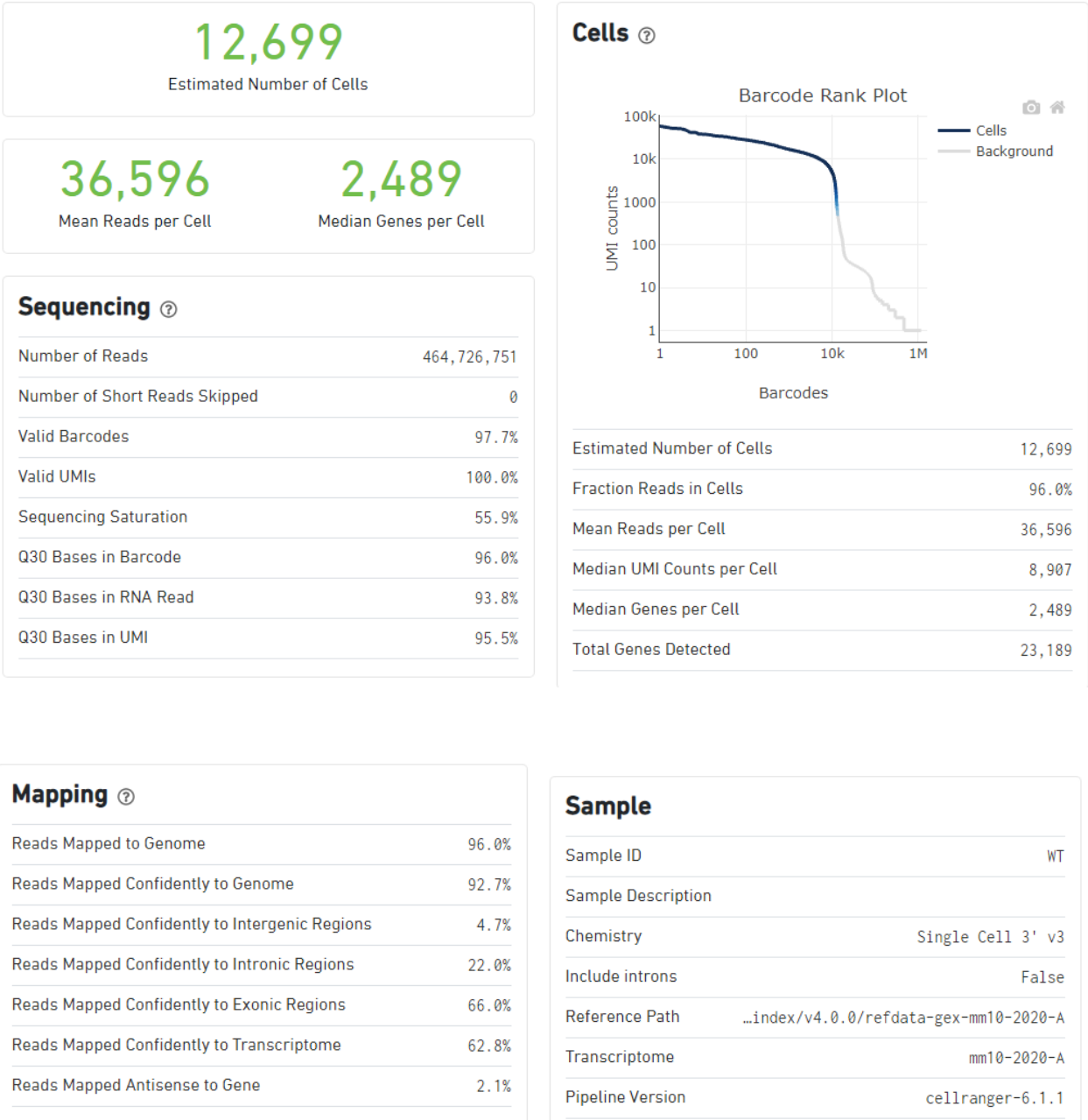

Supplement: Supplementary file 1 [file cells-13-01356-s001.zip › Figure S1.pdf]
